# Breast reconstruction after mastectomy at a comprehensive cancer center

**DOI:** 10.1186/s40064-016-2375-2

**Published:** 2016-07-02

**Authors:** Shahnjayla K. Connors, Melody S. Goodman, Terence Myckatyn, Julie Margenthaler, Sarah Gehlert

**Affiliations:** Division of Public Health Sciences, Department of Surgery, Washington University in St. Louis School of Medicine, St. Louis, MO USA; Division of Plastic and Reconstructive Surgery, Department of Surgery, Washington University in St. Louis School of Medicine, St. Louis, MO USA; Division of Endocrine Oncologic Surgery, Department of Surgery, Washington University in St. Louis School of Medicine, St. Louis, MO USA; George Warren Brown School of Social Work, Washington University in St. Louis, St. Louis, MO USA

**Keywords:** Breast reconstruction, Mastectomy, Breast cancer, Comprehensive cancer center, Disparities

## Abstract

**Background:**

Breast reconstruction after mastectomy is an integral part of breast cancer treatment that positively impacts quality of life in breast cancer survivors. Although breast reconstruction rates have increased over time, African American women remain less likely to receive breast reconstruction compared to Caucasian women. National Cancer Institute-designated Comprehensive Cancer Centers, specialized institutions with more standardized models of cancer treatment, report higher breast reconstruction rates than primary healthcare facilities. Whether breast reconstruction disparities are reduced for women treated at comprehensive cancer centers is unclear. The purpose of this study was to further investigate breast reconstruction rates and determinants at a comprehensive cancer center in St. Louis, Missouri.

**Methods:**

Sociodemographic and clinical data were obtained for women who received mastectomy for definitive surgical treatment for breast cancer between 2000 and 2012. Logistic regression was used to identify factors associated with the receipt of breast reconstruction.

**Results:**

We found a breast reconstruction rate of 54 % for the study sample. Women who were aged 55 and older, had public insurance, received unilateral mastectomy, and received adjuvant radiation therapy were significantly less likely to receive breast reconstruction. African American women were 30 % less likely to receive breast reconstruction than Caucasian women.

**Conclusion:**

These findings suggest that racial disparities in breast reconstruction persist in comprehensive cancer centers. Future research should further delineate the determinants of breast reconstruction disparities across various types of healthcare institutions. Only then can we develop interventions to ensure all eligible women have access to breast reconstruction and the improved quality of life it affords breast cancer survivors.

## Background

 Breast reconstruction after mastectomy has become an important component of the breast cancer care continuum for patients who receive mastectomy (Alderman et al. 2009; Jagsi et al. 2014; Tseng et al. 2010), in part because of its positive effects on psychosocial functioning and quality of life. Breast cancer patients who undergo reconstruction experience less distress, better body image, better sexual function, and higher self-esteem than those who do not undergo reconstruction (Al-Ghazal et al. 2000; Atisha et al. 2008; Harcourt et al. 2003; Rowland et al. 1993; Wilkins et al. 2000). These women also report better physical functioning and less pain (Eltahir et al. 2013). Thus, the long-term benefits that breast reconstruction affords, suggests that equitable access to this procedure optimizes breast cancer survivorship (Alderman et al. 2009).

Although breast reconstruction rates have increased over time, they remain relatively low, ranging from 5 to 42 % in population-based and institution-based studies (Brennan and Spillane 2013; Platt et al. 2011; Wilkins and Alderman 2004). These studies have found that reconstruction rates are significantly lower in African American compared to Caucasian women (Alderman et al. 2009, 2011; Butler et al. 2015; Greenberg et al. 2008; Hershman et al. 2012; Katz et al. 2005; Merchant et al. 2015; Morrow et al. 2005; Reuben et al. 2009; Rosson et al. 2008; Rowland et al. 2000; Tseng et al. 2004; Yang et al. 2013a, b), a disparity that has been attributed to racial differences in access to breast reconstructive services (Agarwal et al. 2011; Enewold et al. 2014; Greenberg et al. 2008; Katz et al. 2005; Offodile et al. 2015; Rowland et al. 1993; Wexelman et al. 2014) secondary to lower socioeconomic status (Alderman et al. 2003; Greenberg et al. 2008; Joslyn 2005; Katz et al. 2005; Kruper et al. 2011a; Offodile et al. 2015; Wexelman et al. 2014) and no or inadequate insurance (Alderman et al. 2006; Bradley et al. 2012; Enewold et al. 2014; Wexelman et al. 2014).

National Cancer Institute-designated Comprehensive Cancer Centers (NCI-CCCs) are tertiary, specialized cancer centers that have higher breast reconstruction rates than those reported by primary healthcare institutions and population-based reports (Brennan and Spillane 2013). This may be because of the more standardized infrastructures imposed by NCI oversight, increased financial resources, higher numbers of reconstructive surgeons focused on breast cancer care, or because surgeons at these institutions are somehow more aware of the psychological impact of breast reconstruction (Greenberg et al. 2011; Reuben et al. 2009). The latter may be due to the cross-disciplinary nature of NCI-CCCs.

It is unclear whether racial disparities in breast reconstruction are reduced in women treated for breast cancer at NCI-CCCs compared to other healthcare institutions, which one would expect if access to care was the sole or most salient issue. Yet, few studies have focused on determinants of breast reconstruction within NCI-CCCs (Christian et al. 2006; Elmore et al. 2012; Greenberg et al. 2011; Sharma et al. 2015; Tseng et al. 2004). It is important to examine breast reconstruction in different types of healthcare institutions to gain a better understanding of the determinants across health care settings (Iskandar et al. 2015). Understanding characteristics that are associated with the receipt of high quality breast cancer care, such as that reported for NCI-CCCs, are essential for identifying and implementing the best practices needed to ensure equitable access to breast reconstruction for all women (Onega et al. 2014). Previous studies at Siteman Cancer Center focused on specific patient groups and utilized smaller sample sizes (Connors et al. 2014; Elmore et al. 2012; Sharma et al. 2015). The purpose of this study was to utilize a large multi-year sample to conduct a comprehensive examination of the rate and determinants of breast reconstruction at Siteman Cancer Center, a NCI-CCC located in Saint Louis, Missouri.

## Methods

### Data source

Siteman Cancer Center is affiliated with the Washington University in St. Louis School of Medicine and BJC HealthCare of St. Louis, a 12-hospital system. We obtained de-identified data for women who received mastectomy for definitive surgical treatment for breast cancer at Siteman Cancer Center between 2000 and 2012. Data were obtained from the Barnes-Jewish Hospital (BJH) Cancer Registry that is maintained by Oncology Data Services (ODS). The Human Research Protection Office (HRPO) and Institutional Review Board (IRB) at Washington University in St. Louis gave this study exempt status.

Data were obtained for 5421 women. The percentage of women of Asian descent was small (n = 120), therefore, we excluded these women from analysis. We also excluded 1147 women who received partial or segmental mastectomies, which are often lumpectomies that rarely receive breast reconstruction. The 1147 women excluded accounted for 1 % of the reconstruction in the original sample. The final sample consisted of 4154 African American and Caucasian women who received mastectomy for definitive breast cancer treatment.

### Independent variables

Sociodemographic variables obtained from the BJC Cancer Registry included age at diagnosis, race, and type of insurance. Age was dichotomized as ≤55 and >55 years and three race categories were present in the Registry, African American, Asian, and Caucasian. Type of insurance was categorized as public (Medicaid, Medicare, Veterans’ Affairs, Indian Health Service) or private [managed care, Health Maintenance Organization (HMO), Preferred Provider Organization (PPO), TRICARE, and fee for service]. Clinical variables analyzed included laterality of the breast tumor (right, left), tumor stage (stage 0/ductal carcinoma in situ (DCIS)-2, stage 3–4), tumor grade (grade 1–2, grade 3–4), estrogen receptor (ER) status (positive, negative), progesterone receptor (PR) status (positive, negative), human epidermal growth factor receptor 2 (HER2) status (positive, negative), triple negative status (no, yes). Triple negative status was only determined in women with expression results for ER, PR, and HER2.

Variables related to breast cancer treatment included type of mastectomy [total/simple, modified radical, radical/extended radical, skin/nipple-sparing, mastectomy not otherwise specified (NOS)], laterality of the mastectomy (unilateral, bilateral), receipt of adjuvant radiation (yes, no), receipt of adjuvant chemotherapy (yes, no), receipt of adjuvant hormonal therapy (yes, no), and receipt of breast reconstruction (yes, no). We also assessed the type of breast reconstruction (tissue expanders/implants, autologous flap, autologous flap and tissue expanders/implants, reduction mammoplasty, scar revision (Y-plasty), reconstruction NOS. The placement of tissue expanders was considered implant-based reconstruction. The primary outcome of interest was receipt of breast reconstruction of the diseased breast after definitive mastectomy for breast cancer treatment.

### Statistical analysis

We first summarized each variable as follows: continuous variables were described by mean and standard deviation and categorical variables were summarized using frequencies and percentages. Independent variables that were significantly associated (p < 0.05) with the receipt of breast reconstruction in Chi squared analysis were examined in unadjusted logistic regression models. Variables with p values <0.1 in the unadjusted models were incorporated into the final multivariable logistic regression model. Adjusted odds ratios, 95 % confidence intervals, and p values were reported for regression analysis. All analysis was performed with Stata SE Version 12 (College Station, TX, USA), statistical significance was assessed as a p value <0.05.

## Results

Sociodemographic and clinical characteristics are displayed in Table 1. The mean age of women in the sample was 55 years old. The majority of women (80 %, n = 3332) were Caucasian. Over half (58 %, n = 1975) of the women in the sample had private insurance, while 42 % (n = 1454) had public insurance. Most women presented with tumors that were stage 0/DCIS, 1, or 2 (81 %, n = 3019) and grade 1 or 2 (59 %, n = 2314). Hormone receptor expression was also favorable; approximately 74 % (n = 2706) and 64 % (n = 2316) of the sample presented with ER positive or PR positive tumors, respectively. Twenty percent (n = 206) of the women presented with HER2 positive tumors and 12 % (n = 128) presented with triple negative tumors. Nearly 80 % (n = 3243) of the sample received unilateral mastectomy as definitive breast cancer surgery. The most common type of mastectomy was total/simple mastectomy (52 %, n = 2148), followed by modified radical mastectomy (40 %, n = 1636). Six percent (n = 267) of the sample received skin-sparing or nipple-sparing mastectomies. Less than half of the women (46 %, n = 1876) received adjuvant chemotherapy and 31 % (n = 1300) received adjuvant radiation. Approximately half of the women (53 %, n = 2164) received adjuvant hormonal therapy.

**Table 1:** Sociodemographic and clinical factors of women treated with mastectomy for breast cancer at the Siteman Cancer Center from 2000 to 2012

Variable	Mean	SD	N
Sociodemographics			
Age at diagnosis	55	13	4154

	Frequency	%	N
Race			
African American	822	20	4154
Caucasian	3332	80	
Age (years)			
<55	2018	49	4154
≥55	2136	51	
Type of insurance			
Private^a^	1975	58	3429
Public^b^	1454	42	
Clinical factors			
Tumor stage			
Stage 0/DCIS–2	3019	81	3709
Stage 3–4	690	19	
Tumor grade			
Grade 1–2	2314	59	3903
Grade 3–4	1589	41	
ER status			
Positive	2706	74	3633
Negative	927	26	
PR status			
Positive	2316	64	3619
Negative	1303	36	
HER2 status			
Positive	206	20	1016
Negative	810	79	
Triple negative cancer			
No	941	88	1069
Yes	128	12	
Laterality of mastectomy			
Bilateral	903	22	4146
Unilateral	3243	78	
Type of definitive mastectomy			
Total/simple	2148	52	4126
Modified radical	1636	40	
Radical/extended modified radical	58	1	
Skin/nipple-sparing	267	6	
Mastectomy NOS	20	0.5	
Received adjuvant chemotherapy			
No	2233	54	4109
Yes	1876	46	
Received adjuvant radiation			
No	2854	69	4154
Yes	1300	31	
Received adjuvant hormonal therapy			
No	1894	47	4058
Yes	2164	53	

*DCIS* ductal carcinoma in situ, *ER* estrogen receptor, *PR* progesterone receptor, *HER2* human epidermal growth factor receptor 2, *NOS* not otherwise specified

^a^Private insurance included: managed care, HMO, PPO, TRICARE, fee for service

^b^Public insurance included: Medicaid, Medicare, Veterans’ Affairs, Indian Health Service

The breast reconstruction rate and types of reconstructive surgery are displayed in Table 2. The breast reconstruction rate of this sample was 54 % (n = 2227). Over half of the women (n = 990) received placement of a tissue expander/implant for reconstruction. Women also underwent reconstruction NOS (39 %, n = 762), autologous flap (7 %, n = 144), and combined autologous flap and placement of a tissue expander/implant for reconstruction (2 %, n = 32). Reduction mammoplasty (0.2 %, n = 3) and scar revision (Y-plasty) (0.5 %, n = 9) were performed in <1 % of women.

**Table 2:** Breast reconstruction rate and type of breast reconstructive surgery of women treated with mastectomy for breast cancer at the Siteman Cancer Center from 2000 to 2012

Variable	Frequency	%	N
Received breast reconstruction			
Yes	2227	54	4152
No	1925	46	
Type of breast reconstruction			
Tissue expander/implant	990	52	1938
Autologous flap	144	7	
Autologous flap and tissue expander/implant	32	2	
Reduction mammoplasty	3	0.2	
Scar revision (Y-plasty)	9	0.5	
Reconstruction NOS	760	39	

*NOS* not otherwise specified

The results of the multivariable logistic regression are displayed in Table 3. In adjusted analysis, the sociodemographic factors that significantly predicted breast reconstruction were age, type of insurance, and race. Women aged 55 years or older where 65 % less likely (aOR 0.35; 95 % CI 0.29–0.42, p < 0.01) to receive breast reconstruction than women under the age of 55. Women with public insurance were 55 % less likely (aOR 0.45, 95 % CI 0.38–0.54, p < 0.01) to receive breast reconstruction than women who had private insurance. African American women were 30 % less likely (aOR 0.70, 95 % CI 0.56–0.87, p < 0.01) to receive breast reconstruction than Caucasian women.

**Table 3:** Multivariable logistic regression of breast reconstruction in women treated with mastectomy for breast cancer at the Siteman Cancer Center from 2000 to 2012 (N = 2809)

Variable	aOR	95 % CI	p value
Age*			
<55	1.00	–	–
≥55	0.35	0.29–0.42	<0.01
Race*			
Caucasian	1.00	–	–
African American	0.70	0.56–0.87	<0.01
Insurance*			
Private^a^	1.00	–	–
Public^b^	0.45	0.38–0.54	<0.01
Tumor stage*			
Stage 0/DCIS–2	1.00	–	–
Stage 3–4	0.60	0.47–0.76	<0.01
PR status			
Positive	1.00	–	–
Negative	0.87	0.73–1.03	0.12
Laterality of mastectomy*			
Bilateral	1.00	–	–
Unilateral	0.37	0.30–0.46	<0.01
Receipt of adjuvant chemotherapy			
No	1.00	–	–
Yes	1.06	0.87–1.30	0.55
Receipt of adjuvant radiation therapy*			
No	1.00	–	–
Yes	0.72	0.59–0.89	<0.01

*DCIS* ductal carcinoma in situ, *PR* progesterone receptor

* Statistically significant variable

^a^Private insurance included: managed care, HMO, PPO, TRICARE, fee for service

^b^Public insurance included: Medicaid, Medicare, Veterans’ Affairs, Indian Health Service

Tumor stage was significantly associated the receipt of breast reconstruction in this sample. Women with stage 3–stage 4 tumors were 40 % less likely (aOR 0.60, 95 % CI 0.47–0.76, p < 0.01) to receive breast reconstruction compared to women with stage 0/DCIS–stage 2 tumors. Treatment factors significantly associated with the receipt of breast reconstruction included laterality of definitive mastectomy and receipt of adjuvant radiation therapy. Women who received unilateral mastectomy were 63 % less likely (aOR 0.37, 95 % CI 0.30–0.46, p < 0.01) to receive breast reconstruction than women who received bilateral mastectomy. Women who received adjuvant radiation therapy were 28 % less likely (aOR 0.72; 95 % CI 0.59–0.89, p = <0.01) to receive breast reconstruction than women who did not receive adjuvant radiation therapy. PR status (aOR 0.87, 95 % CI 0.87–1.03, p = 0.12) and receipt of adjuvant chemotherapy (aOR 0.72, 95 % CI 0.59–0.89, p = 0.55) were not significantly associated breast reconstruction in this sample.

## Discussion

The breast reconstruction rate (54 %) in this sample is high compared to the rates in population-based studies, which range from 5 to 29 % (Platt et al. 2011; Wilkins and Alderman 2004), but is consistent with previously reported rates for this and other NCI-CCCs (Christian et al. 2006; Connors et al. 2014; Elmore et al. 2012; Greenberg et al. 2011; Mitchem et al. 2008; Tseng et al. 2004). Most (52 %) women in this sample received implant-based reconstruction, which is consistent previous studies at Siteman Cancer Center (Connors et al. 2014; Elmore et al. 2012; Mitchem et al. 2008), reflecting the national trend of increasing rates of implant-based breast reconstruction (Albornoz et al. 2012, 2013; Jagsi et al. 2014). Less than 1 % of women received reduction mammoplasty or scar revision (Y-plasty).

Age is one of the most consistent predictors of receiving breast reconstruction (Platt et al. 2011). Our results are consistent with previous studies that report older women are less likely to receive breast reconstruction (Agarwal et al. 2011; Alderman et al. 2003, 2009; Chen et al. 2009; Desch et al. 1999; Elmore et al. 2012; Giladi et al. 2015; Greenberg et al. 2008; Hershman et al. 2012; Iskandar et al. 2015; Jackson et al. 2008; Joslyn 2005; Kruper et al. 2011b; Mahmoudi et al. 2015; Maly et al. 2009; Morrow et al. 2001, 2005, 2014; Polednak 1999; Preminger et al. 2012; Reuben et al. 2009; Rosson et al. 2008; Shippee et al. 2014; Tseng et al. 2010; Yang et al. 2013a). Studies suggest that older women may choose not to have breast reconstruction because they are less disturbed by the prospect of breast loss or more worried about surgical morbidity and complications (Alderman et al. 2003; Morrow et al. 2005; Reaby et al. 1994; Wolfswinkel et al. 2013). Similar to previous studies, women with public insurance were significantly less likely to receive breast reconstruction than those with private insurance (Alderman et al. 2009; Bradley et al. 2012; Christian et al. 2006; Kruper et al. 2011a, b; Mahmoudi et al. 2015; Morrow et al. 2014; Shippee et al. 2014; Yang et al. 2013a). Lack of access to facilities with qualified plastic surgeons and lower payer reimbursement for services may play a role in this relationship (Christian et al. 2006; Kruper et al. 2011a; Preminger et al. 2012). Additionally, for women with public insurance, lower socioeconomic status may serve as a barrier to breast reconstruction because these women must be more attuned to the out-of-pocket costs associated with the procedures, loss of days from work, cost of transportation, and costs of childcare (Katz et al. 2005; Wilkins and Alderman 2004). Siteman Cancer Center has increased its efforts to inform women of available financial, social, and other support services to circumvent these barriers (Fayanju et al. 2013). It is likely that other NCI-CCCs have done the same.

Stage of disease is considered the most predictive clinical factor of receiving breast reconstruction (Platt et al. 2011). In this sample, women with more advanced tumor stages were significantly less likely to receive breast reconstruction. Many studies report women with earlier stages are more likely to receive breast reconstruction (Agarwal et al. 2011; Alderman et al. 2003; Bradley et al. 2012; Chen et al. 2009; Christian et al. 2006; Enewold et al. 2014; Iskandar et al. 2015; Joslyn 2005; Morrow et al. 2001, 2005; Polednak 1999, 2000; Tseng et al. 2004). Although higher stage of disease is not a contraindication for breast reconstruction, women and/or their physicians may prioritize adjuvant treatment above reconstructive procedures (Agarwal et al. 2011; Alderman et al. 2007). Tumor grade did not significantly predict breast reconstruction in this or previous studies (Enewold et al. 2014; Tseng et al. 2010).

The type of breast cancer treatment received correlates with the receipt breast reconstruction (Jagsi et al. 2014). Women who received unilateral mastectomy were significantly less likely to receive reconstruction. This finding is consistent with previous studies (Elmore et al. 2012; Hershman et al. 2012; Iskandar et al. 2015; Jagsi et al. 2014; Joslyn 2005; Preminger et al. 2012; Tseng et al. 2010). Women who have bilateral mastectomies are more likely to be referred to plastic surgeons for breast reconstruction consultations (Preminger et al. 2012) and it has been suggested that women who are highly motivated to receive breast reconstruction may choose bilateral mastectomies, even when it is not clinically indicated, to achieve better symmetry after reconstruction (Tseng et al. 2010). Receipt of adjuvant chemotherapy was not significantly associated with breast reconstruction in our sample. Studies have reported conflicting data on the role of chemotherapy in breast reconstruction. Some studies have reported that receiving neoadjuvant or adjuvant chemotherapy reduces the odds of receiving breast reconstruction (Alderman et al. 2009; Wolfswinkel et al. 2013), while others report that it did not significantly affect the odds of receiving breast reconstruction (Elmore et al. 2012; Enewold et al. 2014; Maly et al. 2009; Morrow et al. 2014). More work is needed to clarify the role of chemotherapy on the receipt of breast reconstruction. Similar to results found in other studies, we found that women who received adjuvant radiation therapy were less likely to receive breast reconstruction (Agarwal et al. 2011; Alderman et al. 2003, 2009; Chen et al. 2009; Enewold et al. 2014; Greenberg et al. 2008; Jagsi et al. 2014; Tseng et al. 2010). Patients who require radiation therapy are less likely to be referred to plastic surgeons for breast reconstruction reflecting concerns on the part of physicians that radiation therapy will negatively impact the results of breast reconstruction (Greenberg et al. 2008; Jagsi et al. 2014). There are currently no uniform guidelines to inform the use of breast reconstruction after radiation therapy and more research is needed to determine the effect of radiation therapy on breast reconstruction outcomes (Elmore et al. 2012; Jagsi et al. 2014).

Even when controlling for sociodemographic and clinical factors, African American women in this sample were 30 % less likely to receive breast reconstruction than their Caucasian counterparts. Although these results are consistent with previous studies (Agarwal et al. 2011; Alderman et al. 2003, 2006; Bradley et al. 2012; Greenberg et al. 2008; Hershman et al. 2012; Jackson et al. 2008; Joslyn 2005; Katz et al. 2005; Kruper et al. 2011a, b, 2013; Lang et al. 2013; Mahmoudi et al. 2015; Morrow et al. 2001, 2014; Offodile et al. 2015; Reuben et al. 2009; Rosson et al. 2008; Shippee et al. 2014; Sisco et al. 2012; Tseng et al. 2004, 2010; Yang et al. 2013a, b), they bolster previous work on the topic by virtue of the relatively high percentage (20 %) of African American women in the sample. Yet our results are contrary to other studies that report no significant contribution of race on breast cancer rates, despite trends towards African Americans having lesser odds of breast reconstruction (Alderman et al. 2009; Chen et al. 2009; Christian et al. 2006; Elmore et al. 2012; Enewold et al. 2014; Iskandar et al. 2015; Kruper et al. 2011b; Maly et al. 2009; Polednak 1999, 2001; Sisco et al. 2012; Wolfswinkel et al. 2013). To our knowledge, only one study has reported that African American women were significantly more likely to receive breast reconstruction than Caucasian women (Giladi et al. 2015). This may have been due to the fact that this study focused on a Medicaid population, with an overall low income. Studies from NCI-CCCs have also produced conflicting results on the role of race on the receipt of breast reconstruction. Race did not significantly affect the odds of receiving breast reconstruction in two studies (Christian et al. 2006; Elmore et al. 2012), while Tseng et al. (2004) found that African American women were 66 % less likely to receive breast reconstruction compared to Caucasian women. The reasons for variations on the role of race on the receipt of breast reconstruction are unclear but may reflect differences in study populations, geographic location, use of different socioeconomic variables, or limited range of patient-related factors (Christian et al. 2006; Wolfswinkel et al. 2013). The results of this and previous studies raise the need for more research to examine racial/ethnic disparities in breast reconstruction across various types of healthcare institutions.

### Limitations

This study has several limitations. It is focused on a single comprehensive cancer center which limits its generalizability to other healthcare institutions or geographical locations. Age, race, and insurance status were the only sociodemographic variables in the BJH Cancer Registry. Therefore, we were unable to fully examine the role of socioeconomic status on breast reconstruction in our analysis and used insurance status as a proxy for socioeconomic status. The Registry lacked information about other determinants of breast reconstruction such as marital status (Agarwal et al. 2011; Chen et al. 2009; Elmore et al. 2012; Hershman et al. 2012; Joslyn 2005) and comorbidities (Chen et al. 2009; Hershman et al. 2012; Reuben et al. 2009; Yang et al. 2013a), including diabetes (Preminger et al. 2012), and obesity (Chen et al. 2009; Christian et al. 2006; Greenberg et al. 2008; Preminger et al. 2012; Wolfswinkel et al. 2013). The strengths of this study included the ability to use a large administrative database to quantify the rate and determinants of breast reconstruction after mastectomy in a NCI-CCC. The large sample of African American and Caucasian women allowed for multivariable regression analysis that incorporated sociodemographic and clinical determinants of breast reconstruction.

## Conclusion

We found that Siteman Cancer Center had a relatively high breast reconstruction rate between 2000 and 2012. After adjusting for sociodemographic and clinical factors, women who were over the age of 55, had public insurance, were treated with unilateral mastectomy, or received adjuvant radiation therapy were significantly less likely to receive breast reconstruction. These findings mirror many of the most common predictors of breast reconstruction previously reported (Brennan and Spillane 2013; Platt et al. 2011; Wilkins and Alderman 2004) and extend the findings of previous studies at Siteman Cancer Center (Connors et al. 2014; Elmore et al. 2012; Sharma et al. 2015) with a larger sample size. Of particular interest was that African American women were 30 % less likely to receive breast reconstruction at this NCI-CCC. The presence of racial disparities in specialized tertiary healthcare institutions suggests that factors outside of access and quality of care may play a role in breast reconstruction disparities. These breast reconstruction disparities have also be reported in Asian, Latina, and Middle Eastern women (Agarwal et al. 2011; Alderman et al. 2003, 2009; Hershman et al. 2012; Iskandar et al. 2015; Katz et al. 2005; Kruper et al. 2011a, b; Kruper et al. 2013; Lang et al. 2013; Mahmoudi et al. 2015; Maly et al. 2009; Offodile et al. 2015; Onega et al. 2014; Shippee et al. 2014; Tseng et al. 2004, 2010; Yang et al. 2013a, 2013b). It is therefore imperative to further examine and address the racial/ethnic disparities in breast reconstruction. Although not considered in this study, patient involvement in decision-making and personal preference may play important roles in breast reconstruction disparities (Greenberg et al. 2008). Few studies focus on these topics among minority women (Rubin et al. 2013). Future research is needed to further delineate the determinants of breast reconstruction disparities across a variety of healthcare institutions, focusing on the role of decision-making and personal preference in minority women. Only then can we develop multilevel interventions to ensure every breast cancer patient has equitable access to breast reconstruction and the improved quality of life it offers to breast cancer survivors.

## Abbreviations

BJH: Barnes-Jewish Hospital
DCIS: ductal carcinoma in situ
ER: estrogen receptor
HRPO: Human Research Protection Office
HER2: human epidermal growth factor receptor 2
HMO: Health Maintenance Organization
IRB: Institutional Review Board
NCI-CCC: National Cancer Institute-designated Comprehensive Cancer Center
ODS: Oncology Data Services
PPO: Preferred Provider Organization
PR: progesterone receptor
NOS: not otherwise specified
